# Costing the economic burden of prolonged sedentary behaviours in France

**DOI:** 10.1093/eurpub/ckac071

**Published:** 2022-08-26

**Authors:** Antoine Noël Racine, Irène Margaritis, Martine Duclos, François Carré, Anne Vuillemin, Christèle Gautier

**Affiliations:** French Ministry of Sport, Pôle Ressources National Sport Santé Bien-Etre, CREPS AURA/Vichy, Vichy, France; French Agency for Food, Environmental and Occupational Health & Safety (ANSES), Maisons-Alfort, France; CHU Clermont-Ferrand, Service de Médecine du Sport et des Explorations Fonctionnelles, Université Clermont Auvergne, INRAE, UNH, Clermont–Ferrand, France; LTSI INSERM, U1099, University of Rennes 1, Rennes, France; Department of Sport Medicine, Pontchaillou Hospital, Rennes, France; Université Côte d’Azur, LAMHESS, Nice, France; French Ministry of Sport, Sport Policy Development Office, National Sport and Health Strategy, Paris, France

## Abstract

**Background:**

There is strong evidence showing that sedentary behaviour time increase the risk to develop several chronic diseases and to premature death. The economic consequences of this risk have never been evaluated in France. The aim of this study was to estimate the economic burden of prolonged sedentary behaviour in France.

**Methods:**

Based on individual sedentary behaviour time, relative risk to develop cardiovascular disease, colon cancer, breast cancer and all-causes of premature mortality were identified. From relative risk and prevalence of sedentary behaviour time, a population attributable fraction approach was used to estimate the yearly number of cases for each disease. Data from the National Health Insurance were used to calculate the annual average costs per case for each disease. Disease-specific and total healthcare costs attributable to prolonged sedentary behaviour time were calculated. Indirect costs from productivity loss due to morbidity and premature mortality were estimated using a friction cost approach.

**Results:**

In France, 51 193 premature deaths/year appear related to a prolonged daily sedentary behaviour time. Each year prolonged sedentary behaviour cost 494 million € for the national health insurance. Yearly productivity loss due to premature mortality attributable to prolonged sedentary behaviour cost 507 million € and yearly productivity loss due to morbidity cost between 43 and 147 million €.

**Conclusion:**

Significant saving and many deaths could be avoided by reducing prolonged sedentary behaviour prevalence in France. To address this issue, strong responses should be implemented to tackle sedentary behaviour, complementary to physical activity promotion.

## Introduction

Last decades, modernization and urbanization of the society have led to change the population’s lifestyle decreasing the physical activity (PA) level and increasing sedentary behaviour (SB) time.^1^^,^^2^ PA is defined as ‘Any bodily movement produced by skeletal muscles that requires energy expenditure’,^2^ whereas SB is defined as ‘any waking behaviour characterized by an energy expenditure ≤ 1.5 metabolic equivalents, while in a sitting, reclining or lying posture’.^3^^,^^4^ In 2020, World Health Organization (WHO) provided for the first time public health guidelines to address health risks of prolonged SB.^1^^,^^2^ The amount of time spent being sedentary should be reduced as much as possible, and it should be replaced by any time and any intensity of PA.^1^^,^^2^

The association of prolonged SB with premature mortality and non-communicable diseases (NCDs) such as cardiovascular disease (CVD), colon cancer, breast cancer is now well established.^5–8^ Despite evidence, there are still limited policy interventions aiming at reducing SB, complementary to health-enhancing physical activity (HEPA) promotion.^9–11^ Yet, meta-analysis showed an interdependent relationship between SB and PA.^12–15^ Thus, meeting the threshold of WHO PA recommendations^1^ is not enough to attenuate detrimental influence of high amount of prolonged daily SB time on premature mortality.^5^ However, recent studies suggested that breaking every hour of sedentary period by few minutes of moderate or vigorous PA has a significant impact on health outcomes.^13^^,^^16^^,^^17^ The difference between physical inactivity, ‘an insufficient PA level to meet present PA recommendations’,^2^ and SB and their respective consequences on health still can be confusing,^3^^,^^18^ especially for policy-makers and the general population.

Moreover, mechanisms and correlates of SB and PA differ.^17^^,^^19–21^ Thus, reducing SB require specific responses, complementary to PA promotion.^9^^,^^22^^,^^23^ Inform policymakers about consequences of health issues, such as physical inactivity and/or SB, and provide evidence to address it, may foster the topic to the policy agenda setting and may help policymakers to their decisions to define and implement policies solutions.^24^^,^^25^

In France based on the INCA 3 study,^26^ the Agency for Food, Environmental and Occupational Health & Safety (ANSES) has highlighted the need to tackle prolonged SB.^27^ From a national representative sample, this study on the lifestyle habits of the French population collected data on SB of 2682 adults through the Recent PA Questionnaire for adults.^26^ According to INCA 3 study, almost 25% of people between 18 and 79 years had a high risk for health conditions with more than 8.6 h of daily SB.^26^ Unlike physical inactivity,^28^ premature deaths due to this SB has never been estimated in France. Likewise, few studies have evaluated the economic burden of physical inactivity,^29^^,^^30^ whereas the economic consequences of SB in France have never been costed. The aim of this study is to estimate premature deaths and the economic burden of prolonged SB in France. In this study, prolonged SB is defined as daily SB time exposure that increasing risk to premature death or to develop NCDs.

## Methods

Direct and indirect economic consequences of prolonged SB in France were estimated in this study. Annual direct healthcare expenditures attributable to prolonged SB in France were calculated using a prevalence-based and population attributable fraction (PAF) approach.^30^^,^^31^ Indirect costs from productivity loss due to morbidity and to premature mortality were estimated using a friction cost approach.^30^^,^^32^

### Quantify direct costs of prolonged SB

Annual direct healthcare expenditures attributable to prolonged SB in France were calculated in four steps: (i) identification and quantification of the increase risk to all-causes premature mortality and to develop NCDs due to prolonged SB; (ii) estimation of the number of heath conditions attributable to prolonged SB using the PAF approach^30^; (iii) calculation of the annual average costs of healthcare expenditures for each disease; and (iv) estimation of the disease-specific and total healthcare costs attributable to prolonged SB.

#### Identification and quantification of the increase risk to all-causes premature mortality and to develop NCDs due to prolonged SB

From meta-analysis or large cohorts, we identified relative risk (RR) of all-causes premature death and to develop NCDs after co-variables adjustments including PA (table 1). To this end, PubMed and Google Scholar databases were used to identify the most suitable studies in order to extract RR with their respective confidence interval (CI). Studies were selected with these following criteria: recent (<10 years) meta-analyses or large cohorts (≥50 000 participants), daily SB time exposure indicated, non-disease participants at baseline, adult participants, studies with RR adjusted for levels of PA. When studies met the criteria, these using accelerometer measurements were privileged instead of studies using questionnaire.

**Table 1:** Mains characteristics of meta-analysis selected

Risks	Study	Participants (*n*)	Thresholds (h/day)	RRs (95% CI)	Adjustments
All-cause premature mortality	Ekelund et al.^5^	36 383	8.6 of SB	1.28 (1.09–1.51)	Sex, age, BMI, socioeconomic position, accelerometer wear time and moderate-to-vigorous-intensity PA
			9.6 of SB	1.71 (1.36–2.15)	
			≥10.8 of SB	2.63 (1.94–3.56)	
CVD	Pandey et al.^8^	740 425	10 of SB	1.08 (1.00–1.14)	Sex, age, BMI, smoking, and CVD risk factors (diabetes and/or hypertension), PA
			≥12.5 of SB	1.14 (1.09–1.19)	
Colon cancer	Morris et al.^6^	430 584	5≥ of TV	1.23 (1.00–1.57)	PA, education, smoking status and intensity, alcohol consumption frequency, family history of colorectal cancer, ever use of hormone replacement therapy (no, yes, unknown), frequency of red and processed meat consumption and stratified by sex, age, Towns deprivation index fifths
Breast cancer	Patel et al.^7^	77 462	≥6 of SB	1.10 (1.04–1.17)	PA, age, race, smoking status, education, alcohol consumption, total energy intake, red/processed meat intake, family history of cancer, prevalent chronic disease, and diabetes.

RRs, relative risk adjusted; CI, confidence interval; SB, sedentary behaviour; CVD, cardiovascular disease; BMI, body mass index; TV, television.

#### Estimation of the number of heath conditions attributable to prolonged SB using the PAF approach

From studies selected in the Step 1, RR adjusted of prolonged SB were extracted with their thresholds in hours per day for all-causes premature mortality and NCDs (table 2). Then, prevalence of prolonged SB in the French population were calculated for each health risk (Supplementary Material S1) using two 2016 collection of open data of the INCA 3 study’ on the lifestyle habits of the French population^33^ and the causes of death of the French National Institute of Statistics and Economic Studies (INSEE).^34^ From these previous calculations, PAF were computed to estimate the yearly number of cases for premature all-causes mortality and for each disease (table 2). For this purpose, the following formula was used^30^^,^^31^:

$$\mathrm{PAF}\;\left(\%\right)=\frac{P_{1}\left(\mathrm{RRs}-1\right)\;}{\mathrm{RRs}}\times 100,$$

**Table 2:** Health risks attributable to prolonged to prolonged SB in France

Health risks	Prolonged SB threshold (h/day)	Prevalence of adults with prolonged SB at baseline (%)	RRs (95% CI)	PAF, % (95% CI)	Number of deaths and NCDs cases attributable to prolonged SB (95% CI)
All-causes premature mortality	8.6 of SB	6.8	1.28 (1.09–1.51)	1.48 (0.56–2.29)	7229 (2735–11 186)
	9.6 of SB	6	1.71 (1.36–2.15)	2.49 (1.58–3.20)	12 163 (7718–15 632)
	≥10.8 of SB	10.5	2.63 (1.94–3.56)	6.51 (5.09–7.55)	31 801 (24 864–36 881)
CVD	10 of SB	15.6	1.08 (1.00–1.14)	1.15 (0–1.91)	55 213 (0–91 702)
	≥12.5 of SB	6.4	1.14 (1.09–1.19)	0.78 (0.53–1.02)	37 449 (25 556–48 972)
Colon cancer	5≥ of TV	9	1.23 (1.00–1.57)	1.68 (0–3.27)	6310 (0–12 620)
Breast cancer	≥6 of SB	51	1.10 (1.04–1.17)	4.64 (1.96–7.4)	31 449 (13 284–50 157)

SB, sedentary behaviour; RRs, relative risk adjusted; CI, confidence interval; PAF, population attributable fraction; NCDs, non-communicable diseases; CVD, cardiovascular disease.

where *P*_1_ is the prevalence of prolonged SB at baseline. RRs is the RR adjusted on prolonged SB comparing with no prolonged SB, adjusted with confounding factors (table 1). For each PAF, 95% CI were computed.

#### Calculation of the annual average costs of healthcare expenditures for each disease

Each year, French National Health Insurance make available data of prevalence and healthcare expenditures of specific disease groupings.^35^ For each disease, data include hospital care expenditures, drug expenditures, physician care expenditures, others health professionals care expenditures, biological tests, transports and daily sickness allowance.^35^ From this open database, 2016 annual average costs per case for each disease was extracted (Supplementary Material S2).

#### Estimation of disease-specific and total healthcare costs attributable to prolonged SB

To estimate total healthcare costs attributable to prolonged SB, we first multiplied yearly number of cases for each disease and their 95% CI by annual average costs of healthcare expenditures. Then, the costs related to each disease were summed to estimate total healthcare costs attributable to prolonged SB in year 2016 (table 3).

**Table 3:** Annual direct healthcare expenditures attributable to prolonged SB in France

NCDs	Prolonged SB threshold (h/day)	Costs of direct healthcare expenditures, € million (95% CI)
CVD	≥10 of SB	317.46 (87.65–481.95)
Breast cancer	≥6 of SB	142.20 (60.03–226.66)
Colon cancer	5≥ of TV	34.69 (0–69.37)

NCDs, non-communicable diseases; SB, sedentary behaviour; CI, confidence interval; CVD, cardiovascular disease; TV, television.

### Quantify indirect costs of prolonged SB

A friction cost approach^32^ was used to estimate productivity losses due to mortality attributable of prolonged SB (≥8.6 h/day). Such as Ding et al*.*,^30^ 3 months of friction period was used. However, we replaced total number of deaths with total number of premature death due to NCDs in France.^36^ Thus, the following formula was used:

Indirect costs of productivity losses = total number of premature deaths due to NCDs in 2016× PAF for all-cause mortality × proportion of deaths occurred at age 15 years or above × employment rates among+ population aged 15 years and above × (Gross Domestic Product per person employed in 2016× 0.25).

To get the proportion of deaths occurred at age 15 years or above, the employment rates among population aged 15 years and above, and the gross domestic product (GDP) per person employed, we used data from INSEE,^34^ the World Bank^37^ and the OECD.^38^ We sensitized friction time using 1.5 months as the lower limit and 4.5 months as the upper limit as recommended by Ding et al.^30^

Productivity losses from workdays lost due to NCDs attributable to prolonged SB were also computed. Data from the study of Vuong et al*.*^39^ were used to estimate the number of workday loss due to NCDs (Supplementary Material S3). Then, the following formula was used to calculate the productivity losses from prolonged SB:Indirect costs of productivity losses = Number of NCDs cases attributable to prolonged SB × mean of workdays lost per year due to the disease × employment rates among population aged 15 years and above × (GDP per person employed in 2016/360).

## Results

### Quantify direct costs of SB

A total of four studies meeting criteria were selected in order to extract RR adjusted of prolonged SB (table 1). Among these studies, only the study of Ekelund et al*.*^5^ used accelerometer to measure SB and to estimate RRs of this risk on all-causes premature mortality according to different thresholds.

The table 2 showed the prevalence of adults with prolonged SB and PAF according to each health risk, as well as the number of deaths and NCDs cases attributable to prolonged SB. In France, 24.9% of adults’ population have a high risk for premature death due to prolonged SB. We estimated that 51 193 (95% CI; 35 317–63 699) premature deaths/year appear related to a daily SB time ≥8.6 h (table 2). In addition, we estimated that 22% of the French adults’ population have a high risk to develop a CVD with ≥10 h of daily SB. This prevalence of prolonged SB might cause 92 662 CVD (95% CI; 25 556–140 674) each year (table 2).

Each year, prolonged SB cost almost 494 million € (95% CI; 147–777) for the National Health Insurance, including 317 million € for CVD, 142 million € for breast cancer, and 34 million € for colon cancer (table 3).

### Quantify indirect cost of SB

Yearly productivity loss due to premature mortality attributable to prolonged SB cost 507 million € (95% CI; 305–636). Whereas yearly productivity loss due to morbidity costs between 43 and 147 million € (table 4).

**Table 4:** Cost of productivity loss due to premature mortality and morbidity attributable to prolonged SB

Health risk	Productivity loss due to premature mortality, € million (95% CI)	
All-causes premature mortality	507.50 (305.12–636.51)	
Health risks	Productivity loss due to morbidity, € million (SE)	
NCDs	without functional limitation due to the disease (S.E.)	with limitation due to the disease (SE)
CVD	31.17 (±720.16)	129.11 (±22.22)
Breast cancer	9.85 (±228.22)	121.67 (±29.05)
Colon cancer	2.11 (±49.04)	26.15 (±6.24)
Total	43.13 (±997.42)	147.82 (±57.51)

CI, confidence interval; SE, standard error; NCDs, non-communicable diseases; CVD, cardiovascular disease.

## Discussion

The results of this study suggest that in 2016, 51 193 (95% CI; 35 317–63 699) deaths might have been avoided if 24.9% of the French adult population had had a daily SB time under the threshold of 8.6 h/day. Moreover, this study provides for the first time a global estimation of the economic burden of prolonged SB in France. Direct and indirect economic consequences of prolonged SB for the French society costed more than 1 billion € in 2016. In the context where the French population is ageing and the prevalence of NCDs will continue to rise,^40^ this economic burden might continue to increase in the forthcoming decades if the prevalence of prolonged SB is not reduced.

For comparison in the UK, Heron et al*.*^41^ estimated that 69 276 deaths could be avoided each year if prolonged SB have been eliminated. There, direct healthcare costs of prolonged SB were estimated at 0.8 billion £ in 2016–17 (almost 0.94 billion € in 2017). In this study, the authors considered prolonged SB as spending at least 6 h of waking time sedentary. This threshold was based on their national health survey, which estimated that 30% of adults on weekdays and 37% of adults on weekend had prolonged SB. Thus, higher direct healthcare costs of prolonged SB in UK were notably due of a higher prevalence of SB than in France. The higher direct costs of prolonged SB in UK could be also explained by the choices of studies’ inclusions in the analysis and by differences between national health systems.

It should be emphasized that our results were probably underestimated. Direct healthcare cost and some parts of indirect cost were only estimated from CVD, colon cancer and breast cancer whereas moderate to strong evidence showed that prolonged SB was associated with several others NCDs.^42^^,^^43^ However, we did not identify recent meta-analyses or large cohorts using daily SB time exposure to estimate the RR for other health outcomes. Some researches have investigated RR by comparing the least sedentary individuals with the most sedentary ones without SB time exposure in hours of day.^42^^,^^43^ Yet, available data on SB of the French population only use daily SB time exposure measurement. Moreover, some large cohorts or meta-analysis studies on the association between SB and NCDs did not take into account some important confounding factors in their RR adjustment. Thus, we did not include these studies in our analysis. For example, Stamatakis et al.^44^ showed that sitting behaviour was not associated with incident diabetes over 13 years, once RR was adjusted on the body mass index at baseline.

Furthermore, some methods also might underestimate the economic consequences of prolonged SB in France. In this study, we used a PAF approach^30^^,^^31^ to estimate direct healthcare costs attributable to prolonged SB. According to Ding et al.^30^ in their economic analyses of the physical inactivity consequences, and comparatively to Carlson et al*.*’s ^45^ study, PAF approach provides lower estimation than more direct approach such as studies which used econometric approach to link data to the risk factor with healthcare expenditures at the individual level.^30^ Moreover, prevalence of prolonged SB daily time in INCA 3 study’ was calculated from data obtained with self-reported questionnaire.^27^ This prevalence and their economic consequences could be underestimated because the use of accelerometer appears more accurate to measure SB than questionnaire which generally underestimates the daily SB time.^46^^,^^47^

On the contrary, indirect costs of productivity due to mortality and to morbidity estimated from a friction cost approach^32^ might be overestimated. This approach considers that in the event of illness or death of a worker there is a loss of productivity for his company for the entire duration of his absence. Although the friction time was sensitized, in the real-world productivity could be partially maintained following a reorganization and optimization of means of production while employee’s absence duration. To estimate productivity loss of absenteeism due to morbidity, we used data of absenteeism of American worker^39^ because we had no data for a French worker. Yet, work absenteeism due of illness may quite differ according the country.^48^

Nevertheless, our results showed that prolonged SB have significant economic impact for the French health system and employers. The workplace could be particularity effective to implement policy interventions aiming to reduce SB and promote PA.^49^^,^^50^ According to a study of Said et al*.*^51^ describing SB of 35 444 French workers, adults spent a mean of 4.17 h of SB per day in work setting. Moreover, when people are sedentary at work, there are more likely to be also outside of work.^51^ Having a global policy to promote active travel as often as possible might also be effective to reduce SB and might generate substantial saving.^52^^,^^53^ French workers spend on average 1.1 h/day in transport sitting.^51^ Moreover, it seems that the development of teleworking increase daily SB time.^54^ There is now strong evidence showing that replacing SB by any duration and any intensity of PA has significant impact on health conditions.^13^^,^^42^ For this reason, policies that need to be implemented to tackle SB will be complementary to HEPA promotion.

This study has certain limitations. Although our analysis was based on RR after PA adjustment, for RR of NCDs we were not able to use studies which investigated dose-response associations between accelerometry measured PA and SB daily time, such as Ekelund et al*.*^5^ for all-causes mortality. Moreover, this study has not included in the analysis all RR studies’ showing an increase risk to develop NCDs. In addition, some RR extracted from large cohorts where association between SB and NCDs were observed in several countries as in France. As described above, indirect costs of productivity loss due to morbidity were computed from data of absenteeism for an American worker. Our study concerned only the French adult population. Yet, data from ANSES^33^^,^^55^ showed that most of the French youth and adolescent not meet the WHO SB guideline.^2^

To conclude, this study shows that many deaths could be avoided by reducing prolonged SB prevalence in France. Moreover, direct healthcare costs attributable to SB-related diseases represent a high economic burden for the French health system. For employers, prolonged SB of workers led to significant productivity loss. To address these issues, strong responses should be implemented to tackle SB, complementary to PA promotion. Further prospective studies with all age cohorts should be developed to analyze the association between PA, prolonged SB, and the risk to develop NCDs from youth to older adults. They would allow to achieve more accurate economic analyses of prolonged SB. Further studies should also investigate economic consequences of prolonged SB in specific groups of population such as people from disadvantaged socio-economic condition in order to help policy-makers to target their policies to reduce SB.

## Supplementary data

Supplementary data are available at *EURPUB* online.

## Funding

This research was supported by the French Ministry of Sport.

*Conflicts of interest*: None declared.

Key points

- Each year, many deaths might be avoided if prevalence of prolonged sedentary behaviour (SB) was reduced.
- In France, direct and indirect economic consequences of prolonged SB costed more than 1 billion € in 2016.
- These results can help policy-makers and employers in their decision to invest in policies aiming to reduce prolonged SB.

## Supplementary Material

ckac071_Supplementary_Data
