# Supplementary material for: Costing the economic burden of prolonged sedentary behaviours in France
Source: Eur J Public Health. 2022 Aug 26;32(Suppl 1):i3–7. doi: 10.1093/eurpub/ckac071 (PMC9421414; doi:10.1093/eurpub/ckac071)
Supplement: ckac071_Supplementary_Data [file ckac071_supplementary_data.docx]

**Supplementary data**

**Supplement 1. Prevalence of prolonged SB in the INCA 3 study and the French population for each health risk.**

| Health risks | Adults sample of INCA 3 study | Prolonged SB^a^ threshold (hours/day) | Prevalence of prolonged SB in the INCA 3 Sample (*n*, %) | Prevalence of prolonged SB in the French population (*n*) |
| --- | --- | --- | --- | --- |
| All-causes mortality | 2 193 | 8,6 of SB 9,6 of SB ≥10,8 of SB | 150 (6.8)  166 (7.6)  230 (10.5) | 3 277 516  3 627 118  5 025 525 |
| CVD^b^ | 2 193 | 10 of SB ≥12,5 of SB | 342 (15,6)  140 (6,4) | 7 472 738  3 059 015 |
| Colon cancer | 2 185 | 5≥ of TV^c^ | 197 (9) | 4 320 232 |
| Breast cancer | 1 164 | ≥6 of SB | 595 (51) | 12 411 985 |

Footnotes: ^a^SB=sedentary behaviour; ^b^CVD=cardiovascular disease; ^c^TV= television.

**Supplement 2. Disease healthcare expenditures in France.**

| NCDs^a^ | Number of cases | Annual average costs, € billion | Annual average costs per case (€) |
| --- | --- | --- | --- |
| CVD^b^ | 4 801 200 | 17.94 | 3 426 |
| Breast cancer | 677 800 | 3.06 | 4 519 |
| Colon cancer | 346 500 | 1.90 | 5 497 |
| Total | 5 825 500 | 22,9 | - |

Footnotes: ^a^NCDs=non-communicable diseases; ^b^CVD=cardiovascular disease**.**

**Supplement 3. Annual average number of workday loss per year due to the disease.**

| NCDs^a^ | Mean of workday loss per year | | |
| --- | --- | --- | --- |
|  | without functional limitation due the disease (S.E^b^) | | without limitation due the disease (S.E) |
| CDV^c^ | 3.03 (±0.07) | 12.66 (±2.16) | |
| Breast cancer | 3.02 (±0.07) | 37.32 (±8.91) | |
| Colon cancer | 3.02 (±0.07) | 37.32 (±8.91) | |

Footnotes: ^a^NCDs=non-communicable diseases; ^b^S.E=standard error, ^c^CVD=cardiovascular disease.

**Supplement 4. Additional references.**

41. Heron L, O’Neill C, McAneney H, Kee F, Tully MA. Direct healthcare costs of sedentary behaviour in the UK. *J Epidemiol Community Health* 2019;73(7):625‑9.

42. World Health Organization. WHO Guidelines on physical activity and sedentary behaviour: Web Annex Evidence profiles: https://www.who.int/publications-detail-redirect/9789240015111 (17 Abril 2022, date last accessed).

43. Physical Activity Guidelines Advisory Committee. Physical Activity Guidelines Advisory Committee Scientific Report. Washington, DC:U.S. Department of Health and Human services, 2018.

44. Stamatakis E, Pulsford RM, Brunner EJ, Britton AR, Bauman AE, Biddle SJ, et al. Sitting behaviour is not associated with incident diabetes over 13 years: the Whitehall II cohort study. *Br J Sports Med* 2017;51(10):818‑23.

45. Carlson SA, Fulton JE, Pratt M, Yang Z, Adams EK. Inadequate Physical Activity and Health Care Expenditures in the United States. *Prog Cardiovasc Dis* 2015;57(4):315‑23.

46. Ferrari GL de M, Kovalskys I, Fisberg M, Gómez G, Rigotti A, Sanabria LYC, et al. Comparison of self-report versus accelerometer – measured physical activity and sedentary behaviors and their association with body composition in Latin American countries. *Plos One* 2020;15(4):e0232420.

47. Healy GN, Clark BK, Winkler EAH, Gardiner PA, Brown WJ, Matthews CE. Measurement of Adults’ Sedentary Time in Population-Based Studies. *Am J Prev Me*d 2011;41(2):216‑27.

48. Jakovljevic M, Malmose-Stapelfeldt C, Milovanovic O, Rancic N, Bokonjic D. Disability, Work Absenteeism, Sickness Benefits, and Cancer in Selected European OECD Countries—Forecasts to 2020. *Front Public Health* 2017;5:23.

49. Braun A, Franczukowska AA, Teufl I, Krczal E. The economic impact of workplace physical activity interventions in Europe: a systematic review of available evidence. *Int J Workplace Health Manag* 2022.

50. Lam K, Baurecht H, Pahmeier K, Niemann A, Romberg C, Biermann-Stallwitz J, et al. How effective and how expensive are interventions to reduce sedentary behavior? An umbrella review and meta-analysis. *Obes Rev* 2022;23(5):3422.

51. Saidj M, Menai M, Charreire H, Weber C, Enaux C, Aadahl M, et al. Descriptive study of sedentary behaviours in 35,444 French working adults: cross-sectional findings from the ACTI-Cités study. *BMC Public Health* 2015;15(1):379.

52. Cavill N, Kahlmeier S, Rutter H, Racioppi F, Oja P. Economic analyses of transport infrastructure and policies including health effects related to cycling and walking: A systematic review. *Transp Policy* 2008;15(5):291‑304.

53. Barban P, de Nazelle A, Chatelin S, Quirion P, Jean K. Quantifying the health benefits of physical activity due to active commuting in a French transition scenario: a health impact assessment of the negaWatt scenario. *Public and Global Health* 2022.

54. Escalon H, Deschamps V, Verdot C. Physical activity and sedentary behaviour of French adults during the COVID-19 lockdown: an overview of prevalence and perceived evolutions (CoviPrev, 2020). *Bull Epidémiol Hebd* 2021;2(13).

55. Agence Nationale de Sécurité Sanitaire de l’Alimentation, de l’Environnement et du Travail (ANSES). Actualisation des repères du PNNS–Révisions des repères relatifs à l’activité physique et à la sédentarité: https://www.anses.fr/fr/content/plus-d%E2%80%99activit%C3%A9-physique-et-moins-de-s%C3%A9dentarit%C3%A9-pour-une-meilleure-sant%C3%A9 (17 Abril 2022, date last accessed).
